# Stop and Smell the Pollen: The Role of Olfaction and Vision of the Oriental Honey Buzzard in Identifying Food

**DOI:** 10.1371/journal.pone.0130191

**Published:** 2015-07-15

**Authors:** Shu-Yi Yang, Bruno A. Walther, Guo-Jing Weng

**Affiliations:** 1 Institute of Wildlife Conservation, College of Veterinary Medicine, National Pingtung University of Science and Technology, Pingtung, Taiwan; 2 Master Program in Global Health and Development, College of Public Health and Nutrition, Taipei Medical University, Taipei, Taiwan; University of California, San Diego, UNITED STATES

## Abstract

The importance of olfaction for various avian behaviors has become increasingly evident. So far, the use of olfaction for food detection among raptors has only been demonstrated for *Cathartes* vultures. The Oriental honey buzzard (*Pernis orientalis*) is a resident and migrant in Taiwan and regularly forages in apiaries. One of its foods in apiaries is yellow pollen dough, a softball-sized mixture of pollen, soybeans, and sugar that beekeepers provide as a supplementary food for bees. Given that pollen dough is not similar to any naturally occurring food, we hypothesized that buzzards identify the dough’s nutritious contents using olfaction, perhaps in combination with vision. Using a series of choice experiments in which individuals could choose between two doughs, we showed that (1) buzzards almost unerringly chose pollen-containing over pollen lacking doughs when otherwise the doughs were identical in size, shape, and yellow color; (2) buzzards always preferred yellow over black or green doughs if both doughs contained pollen; (3) buzzards still preferred pollen-containing over pollen-lacking doughs when both doughs were black, but at a lower rate than in (1). We statistically excluded the possible influences of the doughs’ relative brightness or of repeat visits by the same individuals. Our experiments thus suggest the use of a ‘multi-modal foraging strategy’ among buzzards whereby olfaction and vision are likely to be both used in identifying food at close distances. We also estimated the olfactory receptor gene repertoire size in the buzzard’s genome which is almost five times as large as that of three other raptor species. Therefore, olfaction is likely of far greater ecological importance to this species than to other raptor species. We suggest that olfaction should be considered in the design of behavioral and genetic studies to better understand the use of multiple senses in avian behaviors.

## Introduction

A long-held assumption was that birds rely mostly on vision and hearing because they have been assumed to have a relatively limited sense of smell. However, research in recent decades has shown that at least some bird species, e.g., kiwis [1, 2], pigeons [3], tube-nosed seabirds [4], and *Cathartes* vultures [5], have quite an acute sense of smell [6, 7]. Birds use olfaction for navigation [8, 9], food detection [10, 11], nest location and material [12, 13] and possibly predator avoidance [14–16] and intraspecific communication and recognition [17–20].

The question arises how birds combine multiple senses to enhance their success in specific behavioral tasks. Some cases of multi-sense integration in birds have been reported. For example, procellariiform species initially use olfaction to detect a prey’s location and then use vision for prey capture [21, 22]. Japanese quails (*Coturnix coturnix japonicus*) and domestic chickens (*Gallus gallus domesticus*) use vision and olfaction in the detection of aposematic insects [23–27]. Therefore, olfaction plays an important role in some specific behavioral tasks of birds. Here, we approach this question by testing the use of olfaction by a raptor species and examining the interaction of vision and olfaction in food identification.

The Oriental honey buzzard (*Pernis orientalis* [28] or *Pernis ptilorhynchus orientalis* [29]) inhabits woodlands of various climatic types, preferring broad-leaved forests, and has a large distribution which covers the Indian subcontinent and large parts of East Asia. During the winter, several thousand migrants or winter visitors come to Taiwan [28] which far outnumber the breeding population (see also Methods). Some of these individuals can be found foraging in or around apiaries. They typically perch in the canopy near or above an apiary before they decide to come to the ground to feed, among others, on honey combs and larvae discarded by beekeepers (Severinghous & Huang [30, 31]; our own observations). In addition, they consume the so-called ‘pollen dough’ (also called pollen balls), a mixture of pollen, soybeans and sugar, which beekeepers provide as a supplementary food for bees during the winter months.

To our knowledge, *Cathartes* vulture species are so far the only diurnal raptors which were conclusively shown to use olfaction for food detection [5, 32–35]. Pollen doughs usually look similar to a yellow softball. Given that pollen dough is not a naturally occurring food or looks similar to any naturally occurring food, we hypothesized that Oriental honey buzzards might detect one or more ingredients in the dough using their olfaction. Since pollen doughs prepared by beekeepers are always yellow, it may also be the yellow color which attracts the buzzards. To test these hypotheses, we staged a series of two-choice experiments in the field which were meant to demonstrate that (1) buzzards can distinguish between doughs containing or lacking an ingredient (either pollen, sugar, or soybean) using olfaction, and (2) buzzards are also influenced by the dough’s color.

To test for the physiological basis of olfaction, we also estimated the olfactory receptor (OR) gene repertoire size in the genome of the Oriental honey buzzard. The number of different scents that a species can identify is related to the total number of OR genes in its genome [36, 37]. Among bird species, the total number of OR genes was also found to correlate with olfactory capability [38]. Moreover, the proportion of functional OR genes which are gene sequences without an associated stop codon is assumed to reflect how much a species has relied on olfaction during the course of its evolution [37, 39]. Among the OR genes of birds, the clade group γ-c is a shared characteristic of all avian genomes [38]. Therefore, we adopted a PCR-based approach [38] to estimate the number of functional and non-functional OR genes in the clade γ-c group and the non-γ-c group of the Oriental honey buzzard and compared these results to those reported for other bird species.

## Methods

### Ethics statement

We adhered strictly to the “Guidelines for the use of animals in research” published in *Animal Behaviour*. The research proposal was approved by the Institutional Animal Care and Use Committee at the National Pingtung University of Science and Technology (Approval no. NPUST-IACUC-101-057). Because this research did not involve any trapping, handling, sacrificing, collecting or physical contacting of any parts or whole animals, permission was not required from any governmental agencies according to the Article 18 of the Wildlife Conservation Act of Taiwan. The research was observational in nature, conducted in private properties and caused no undue harm to the Oriental honey buzzard. All the land owners were aware of our research and permitted us to conduct the research in their properties. To avoid possible disturbances from observers, we either videotaped or observed the birds in a blind using binoculars. Although all the raptor species in Taiwan are categorized as ‘protected species,’ the Oriental honey buzzard is not endangered and our observations did not disturb or physically contact the birds as mentioned above. Detailed methods of the field experiments are provided in the section ‘Choice experiments’ below.

### Choice experiments

**Fig 1:**
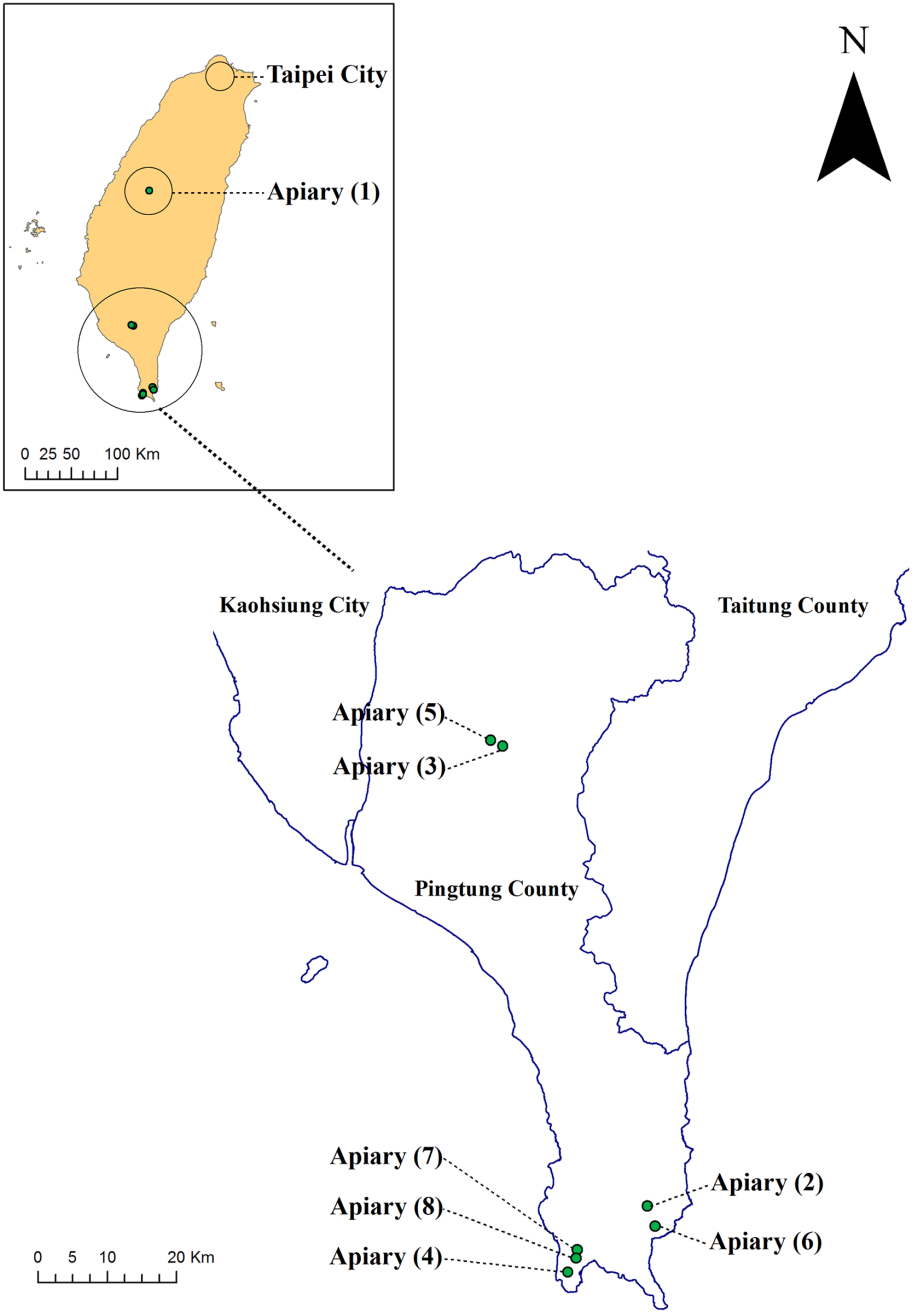
Distribution of eight apiaries in central and southern Taiwan where our 216 two-choice experiments were conducted. See S1 File for names and coordinates of the apiaries.

A total of 216 two-choice experiments were conducted in eight apiaries (1: Jiufengshan, 2: Jiulongshan, 3: Niujiaowan, 4: Hejie, 5: Zhonglin, 6: Manzhou, 7: Longquan, 8: Guanshan) in central and southern Taiwan (Fig 1, S1 File) from 8:00–16:00 hours during three winters from 2010 to 2012 and one month of the breeding season, namely May 2012, when buzzards were still present in one apiary.

All the ingredients for making our doughs (except the dyes) were purchased from one beekeeper to ensure that our doughs were identical to those usually offered in the apiaries. We maintained the ingredients and formula of making doughs (see below) in all experiments to ensure that doughs were identical in each trial.

In all of our two-choice experiments, two softball-sized doughs of identical size (800 g) and shape were simultaneously presented about 50 cm apart on a white canvas on the ground of an open space inside the apiary; their placement on either side of the canvas was randomized. An open space was any reasonably sized space without any high vegetation which was not used by beekeepers for the operation of their apiary.

We defined a visit of these two doughs as any individual appearing within 30 cm from the doughs with its head bowed down towards the doughs but not actually touching the doughs. A non-visit was defined as the two doughs not being visited during the entire day of the experiment. To maximize data collection, visits were recorded by either (1) direct observations or (2) a continuously recording video camera (44.2% and 55.8% of the total of 113 visits, respectively).

If an individual fed on a dough one or several times, we recorded this as one foraging event. Therefore, when a buzzard visited a choice experiment, only zero or one event could be recorded for each dough but an experiment could generate a total of zero, one, or two foraging events because two doughs were present. However, zero was never actually recorded because each visit resulted in at least one event (see Results). Once a visit had taken place and the individual or individuals had left, the old doughs were removed and two new doughs were provided to begin another experiment if one of us had done the observations. However, if the observation was done by camera, then the doughs were not renewed during that day.

#### Experiment 1: varying ingredients with constant color yellow

In this experiment, we varied the food ingredients while we kept the dough’s color a constant yellow. One dough contained all three ingredients, namely pollen, soybean and sugar, which is the original pollen dough mixture usually provided by the beekeepers. In each experiment, an original pollen dough was either paired with a dough lacking pollen (experiment 1a), a dough lacking soybean (experiment 1b), or a dough lacking sugar (experiment 1c) (Table 1). For the original pollen dough, pollen, soybean, sugar and water were mixed in a 1:6:2:1 proportional mix. For the other doughs, the relative proportions were always maintained; however, if soybean was lacking, it was replaced by flour to maintain the solidity of the dough. We thus presented the buzzards with three sets of paired comparisons with the presence or absence of each ingredient being the treatment factor in each set. Both doughs were dyed yellow using an artificial food colorant which is the typical color of the pollen dough usually used by beekeepers. The dye was approximately 0.2% of the total weight of the dough; therefore, we assume that the smell of the dye was negligible compared to the smell of the other ingredients. Furthermore, using food dyes is a common method in olfactory research (e.g., [23, 25, 27]).

**Table 1:** Numbers of repetitions and visits of each experiment.

Experimental identifier	Treatment	Color of doughs	N_r_	N_v_	N_e_	N_i_	N_ind_
1a	Pollen	Yellow	30	27 (90%)	29	15	≥14
1b	Soybean	Yellow	7	7 (100%)	14	2	≥1
1c	Sugar	Yellow	40	29 (73%)	53	12	≥7
2	Color	Yellow, black/green	44	23 (52%)	23	8	≥8
3	Pollen	Black	95	27 (28%)	30	14	≥10
Total			216	113 (52%)	149	51	≥40

**Fig 2:**
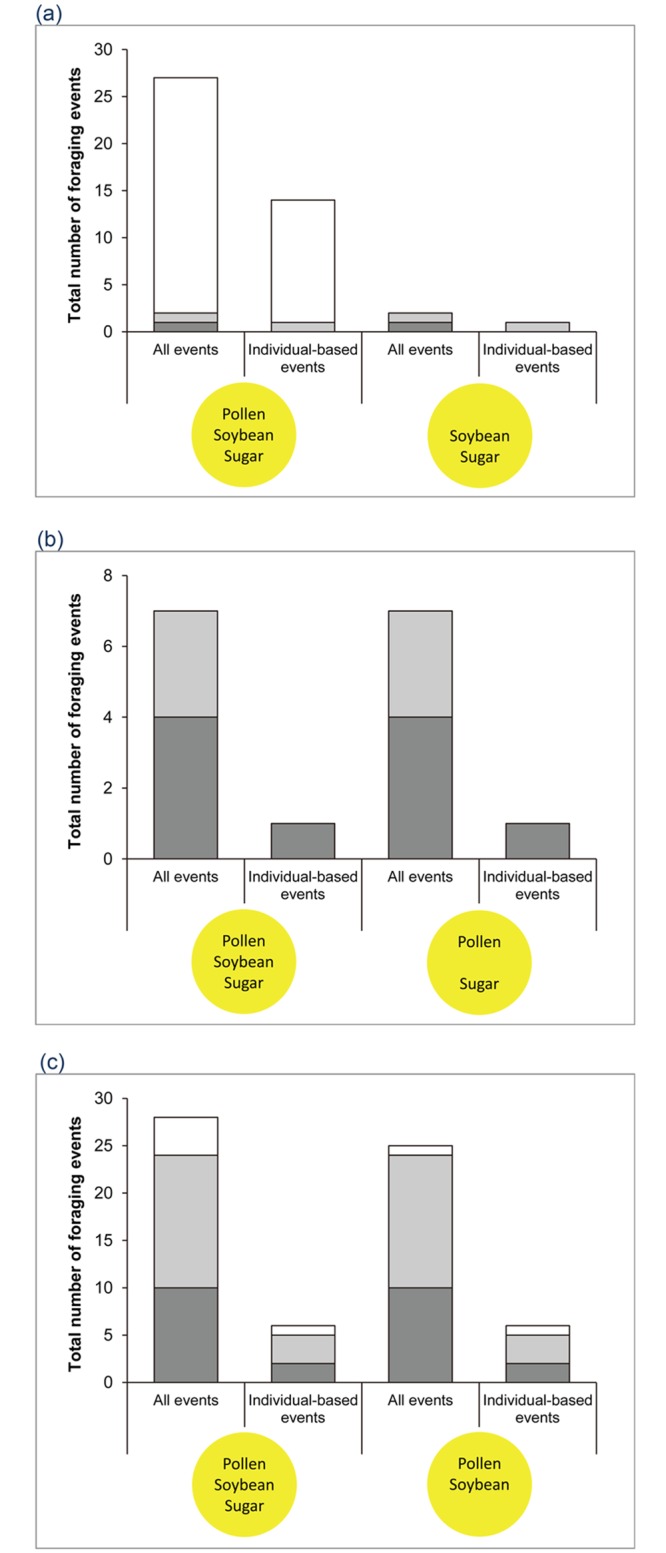
Number of foraging events in experiment 1 where the treatment is presence or absence of (a) pollen, (b) soybean and (c) sugar in yellow doughs (Table 1). Open bars: number of events feeding on only one dough; light grey bars: number of events feeding on both doughs, whereby the first chosen dough contained all three ingredients; dark grey bars: number of events feeding on both doughs, whereby the first chosen dough contained only two ingredients.

**Fig 3:**
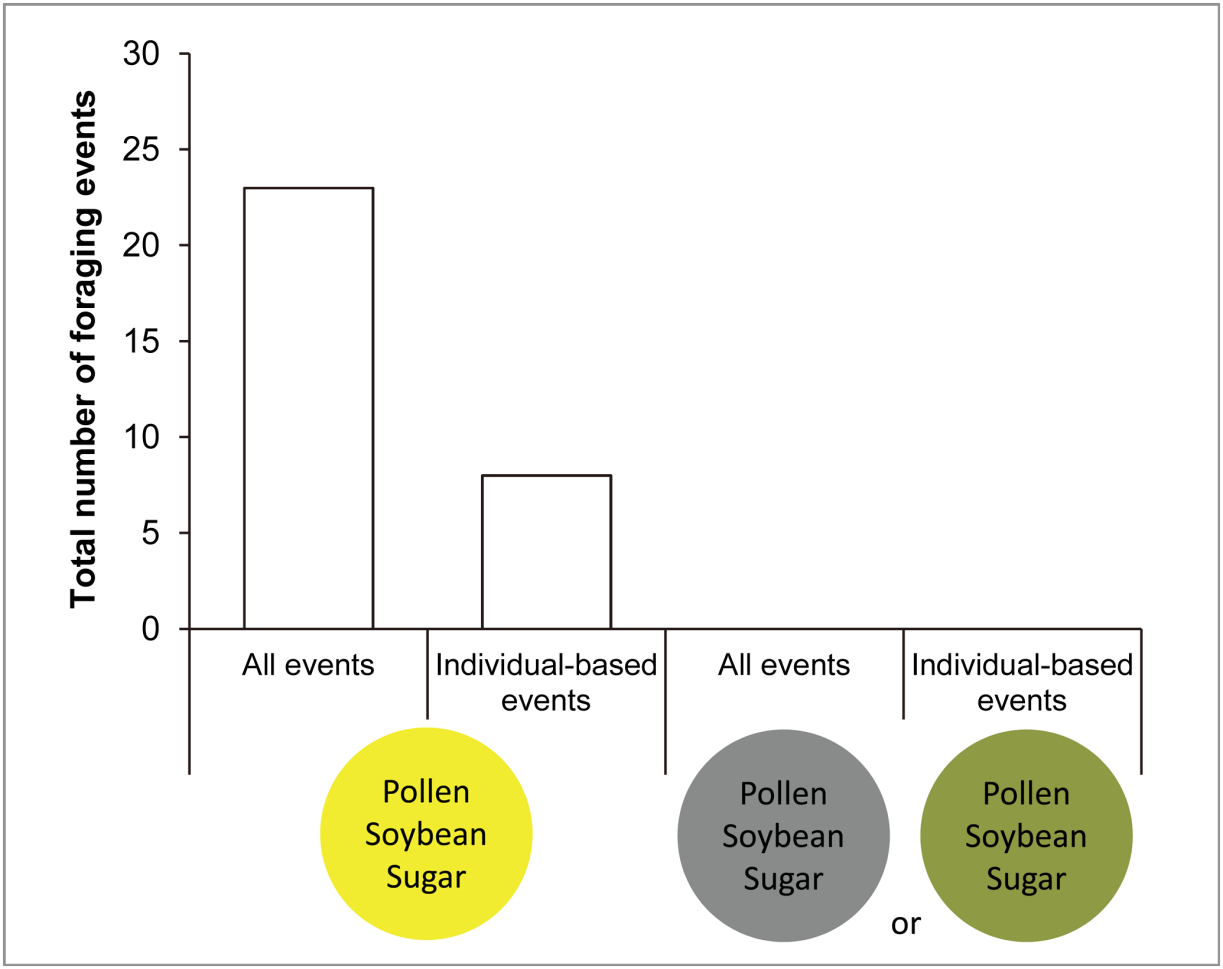
Number of foraging events in experiment 2 where the treatment is color (yellow versus black or green) in doughs containing pollen (Table 1). Open and solid bars as in Fig 2.

**Fig 4:**
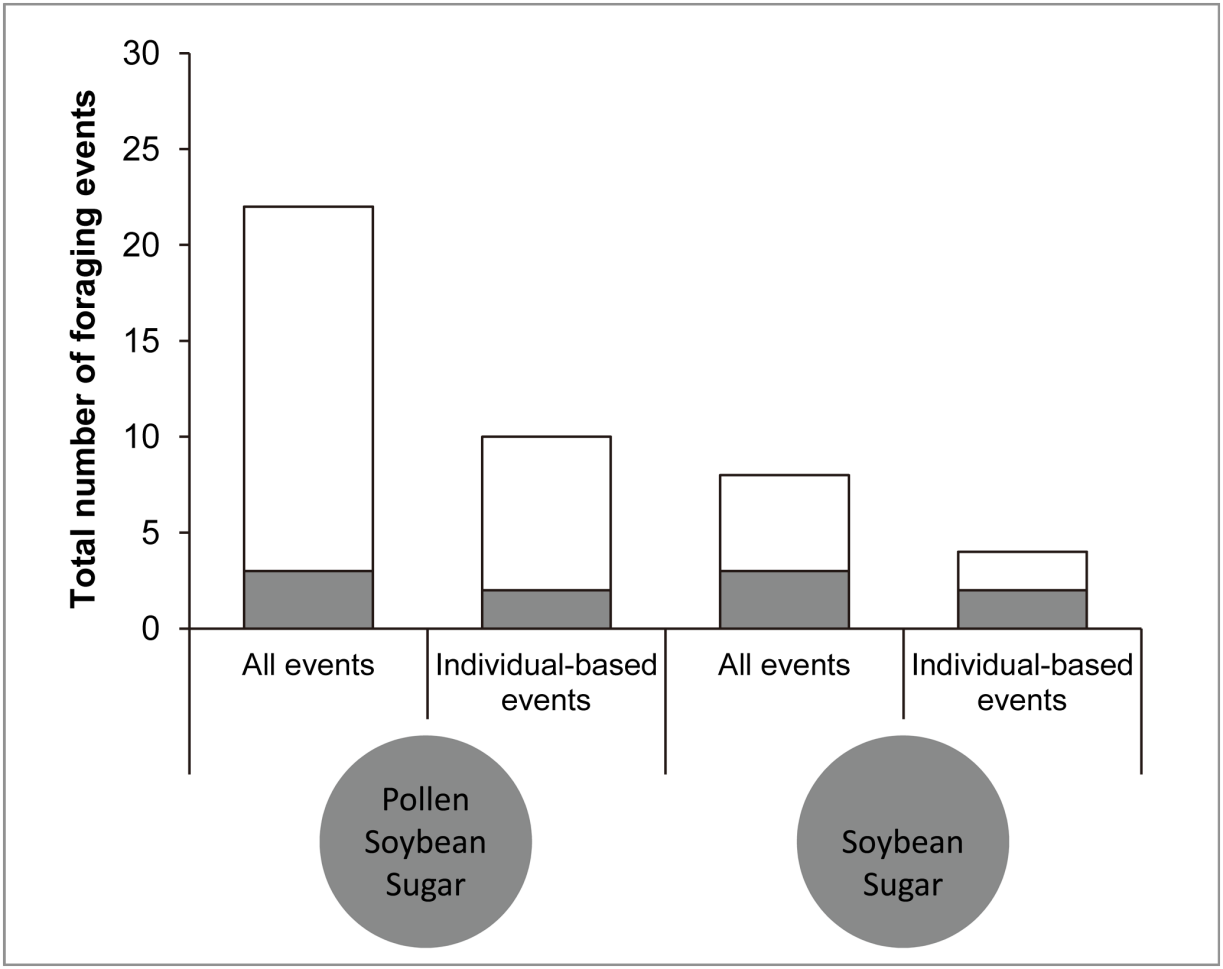
Number of foraging events in experiment 3 where the treatment is presence or absence of pollen in black doughs (Table 1). Open and dark grey bars as in Fig 2, whereby dark grey bars means that the first chosen dough contained only two ingredients.

Column 1: for each experiment’s identifier, see Methods and Figs 2–4. N_r_, number of repetitions of each experiment which we carried out; N_v_, number of experiments visited by Oriental honey buzzards (with the percentage in parentheses); N_e_, number of all foraging events; N_i_, number of individual-based foraging events; N_ind_, number of identified individuals which is hence the minimum number of individuals for testing our hypotheses.

#### Experiment 2: varying color with constant ingredients

We varied the dough’s color while we kept the dough’s ingredients constant. One dough was dyed yellow and the other one was dyed black (present during 18 visits) or green (present during 5 visits) using artificial food colorants, but both doughs contained the original pollen dough mixture as in experiment 1 (Table 1). We used two colors (black and green) because individuals may have aversive reactions to one particular color. However, our results showed no difference between the colors, and therefore we lumped the results for black and green doughs.

#### Experiment 3: varying pollen with constant color black

Since the buzzards disproportionately selected pollen-containing doughs (see Results), we finally varied only the ingredient pollen while we kept the dough’s color a constant black using an artificial food colorant (Table 1). It was thus a repetition of experiment 1a, but with a different color.

### Measuring the dough’s spectra

To ensure that the doughs’ reflected spectra were different for different colors but very similar for the same color, we measured the spectra of light reflected by doughs from 290 nm to 1100 nm of wavelength, thus covering the entire ultraviolet, visible and infrared range. A USB4000 spectrometer (Ocean Optics, Dunedin, Florida, USA) was used for ultraviolet light (wavelength < 400 nm) and a USB2000 spectrometer for visible light (wavelength > 400 nm).

Since we did not detect any difference in the overall shape of the spectra among the four types of yellow doughs and the two types of black doughs (see Results), the only possible factor which might confound our results was the differential brightness (i.e., luminance) of the doughs. However, light conditions, including brightness, vary constantly in natural environments. Therefore, it is unlikely that birds consistently change their food choices simply because the brightness of the food item changes. The relative brightness of two doughs in the same experiment, on the other hand, might affect the buzzards’ choices. Therefore, we compared the relative brightness of the two doughs and categorized each of the two doughs as either darker or brighter within an experiment. To avoid the influence of color, we only used experiments 1 and 3 which had constant colors for this test.

### Ensuring independence of foraging events

Since it is a reasonable assumption that individual buzzards learnt about the food sources and repeatedly visited our experiments, we used two lines of evidence to ensure that we did not repeatedly observe the same individuals:

1. Previous research from our study area had demonstrated that of 61 individuals ringed during the winter seasons from Oct 2007 to Nov 2012, only two were recaptured or resighted in the following winters; thus, fifty-nine individuals (96.7%) never showed up again in the study area (Yang and Severinghaus, unpublished data). Furthermore, Oriental honey buzzards are known to move around the entire country during winter [40]. We thus assumed that most individuals visiting our experiments were migrating visitors that made a stopover in Taiwan on their way to the Philippines instead of permanent local residents. We therefore regarded individuals which appeared in different winters as distinct individuals.
2. Within winters, we ensured the independency of foraging events by allowing only one visit to each experiment by each identified individual during each winter. Some individuals were individually identifiable using a combination of plumage, age and sex. The plumage of the head, face, throat, chest and belly varies from completely pale yellow to light brown, dark brown or black, with or without variable patterns of stripes on the face, throat, chest and belly. The male has red irises, two relatively broad, dark tail bands, and black trailing edges to the wings. In contrast, the female has yellow irises, multiple thinner tail bands, and no prominent trailing edges to the wings. Furthermore, first-year juveniles have a bright yellow cere, but after the first year, black dots develop on the cere until the cere is completely black, which may take several years. If we positively identified an individual to visit an experiment more than one time using the above characters, we did not count the results from repeated visits unless it behaved differently (see Results).

Foraging events selected according to these two restrictions were called ‘individual-based events.’ We then analyzed our data (1) using all events produced by all observed individuals and (2) using only the individual-based events as defined above.

### Statistical analysis

In those few cases when the buzzards foraged on both doughs, the foraging on one dough may not be statistically independent from the foraging on the other dough because taste may then become involved in the selection of doughs. We therefore used the dough chosen first in each visit to test whether buzzards selected any particular type of dough. We applied a generalized linear model (GLM) using the GENMOD procedure in SAS 9.4 (SAS Institute Inc., Cary, NC, USA) to model the probabilities of foraging first on the original yellow pollen dough (experiments 1 and 2), the black dough with all three ingredients (experiment 3), and the darker dough in all three experiments (effect of relative brightness). Since we considered only the first chosen dough, the response variables follow a binomial distribution. A logit function was used to link the response variables above with the independent variables of either ingredient (experiments 1 and 3), color (experiment 2), or relative brightness (all experiments).

However, in experiment 2, buzzards exclusively fed on yellow doughs (see Results), so that the response variable has only one possible state and thus eliminates the use of a GLM. If buzzards did not differentiate between the colors of the two doughs, we would expect a 50:50 distribution of foraging events on the two doughs. We therefore used a Chi-square test with one degree of freedom to examine if the frequencies of these events were biased away from a 50:50 probability.

To test for an interaction between olfaction and vision, we used a GLM to model the probability of feeding first on the pollen-containing dough in experiments 1a and 3, with the dough’s color as the independent variable. In this case, we assumed that if vision did not in some way interact with olfaction, the dough’s color would not have a significant effect on the probability of feeding first on the pollen-containing dough.

Finally, we modeled the visitation rate (i.e., the proportion of replicated experiments visited by buzzards, cf. Table 1) with a GLM, with the proportion of yellow doughs provided in each respective experiment (namely, 1 in experiment 1, 0.5 in experiment 2, 0 in experiment 3, Table 1) as the independent variable. If vision was involved in the doughs’ detection, the proportion of yellow doughs would have a significant effect on the visitation rate.

### Olfactory receptor gene analysis

Blood samples collected during the study of Severinghous and Huang [31] and stored in 99% alcohol at -20°C were used for genomic DNA isolation with a commercially available kit (FavorPrep Blood/Cultured Cell Genomic DNA Extraction Mini Kit, Favorgen Biotech, Pingtung, Taiwan). We used degenerated primer combinations designed by Steiger et al. [38] to amplify the partial γ-c and non-γ-c OR genes in PCR with a final volume of 20 μl, containing 1 μg of genomic DNA template, 0.2 μM of each primer, 1.25 U Taq DNA polymerase (Thermo Scientific, MA, USA), 100 μM dNTPs and 0.75 mM Mg^2+^ in a GeneAmp PCR system 9700 (Applied Biosystems, CA, USA) with the following thermocycling parameters: 94°C/10 min; 94°C/15 s, 45°C/30 s, 72°C/20 s, 40 cycles; 72°C/1 min; and 4°C/hold. PCR products were separated in 1.8% agarose gels before ligation to a cloning vector (yT&A Cloning Vector, Yeastern Biotech, Taipei, Taiwan). Plasmids were purified from transformed DH5α colonies using a commercial kit (FavorPrep Plasmid DNA Extraction Mini Kit, Favorgen Biotech, Pingtung, Taiwan), and the sequencing was then conducted by the Institute of Biomedical Sciences, Academia Sinica, Taipei, Taiwan. Electropherograms were inspected using the software Chromas which was used to delete PCR primer sequences. Sequences were then examined using the BLASTX search in the NCBI’s database (http://www.ncbi.nlm.nih.gov/) to eliminate any sequences which did not return a ‘best hit’ within a vertebrate OR gene.

To identify non-functional OR genes, sequences were then checked using two established but separate techniques: (1) the open reading frame finder at the NCBI website and (2) the Molecular Toolkit at the Colorado State University web site (http://arbl.cvmbs.colostate.edu/molkit/). A sequence was considered as non-functional when all the above checks demonstrated the presence of a stop codon in the sequence. We then continued with the procedures and software described in detail in Steiger et al. [38] to identify those sequences which originated from a single OR gene in order to estimate the total number of OR genes in the genome of the Oriental honey buzzard.

We also collated OR gene repertoire sizes for other birds species from Steiger et al. [38], Warren et al. [41], Zhan et al. [42] and Doyle et al. [43]. While the number of OR genes was estimated based on degenerated PCR in Steiger et al. [38] and this study, genome search was used in the other three studies. However, Steiger et al. [38] compared results of these two techniques using the domestic chicken and concluded that degenerated PCR provided a reasonably reliable estimate of the total number of OR genes.

## Results

Experiments 1a, 1b, 1c, 2 and 3 were repeated 30, 7, 40, 44 and 95 times, respectively (Table 1). We repeated experiment 3 the most times because its visitation rate was the lowest (28%). The 216 repetitions of our five experiments resulted in a total of 113 visits with 149 events and 51 individual-based events which were based on at least 40 different individuals (Table 1). Each visit resulted in at least one event.

Of those individuals that we were able to identify individually, eight individuals visited the same experiment twice, but only two of these individuals foraged on different kinds of dough during their two visits of experiment 3. Therefore, we scored these differing foraging events by these two individuals as two individual-based events, as defined above. The other six individuals did not change their choice during their two visits to same experiment, and we thus scored them as only one individual-based event.

### Experiment 1: varying ingredients with constant color yellow

This experiment showed a clear difference between the three ingredients. When the treatment factor was pollen, buzzards strongly preferred pollen-containing doughs. Buzzards exclusively chose pollen-containing doughs in 25 out of 27 visits (92.6%) when all events were considered and 13 out of 14 visits (92.9%) when only the individual-based events were considered (Fig 2a). In either case, buzzards never chose only the pollen-lacking dough, and tried both doughs only twice and once when all events and all individual-based events were considered, respectively. The P-values were significant for choosing a pollen-containing dough when all events were considered, and very close to the significance value of P = 0.05 when only individual-based events were considered (Table 2). On the other hand, no significant effect was detected when the treatment factor was soybean (Table 2, Fig 2b) or sugar (Table 2, Fig 2c), with almost all individuals choosing both pollen-containing doughs (although experiment 1b has a rather low sample size).

**Table 2:** GLM models for the effects of ingredient, color, interaction of ingredient and color, and relative brightness on dough chosen first as well as the effect of the dough’s color on the buzzards’ visitation rate.

Experimental identifier, treatment and data set	Independent variable	Parameter estimate	SE	Chi-square	P
1: Ingredient (with constant color yellow)					
All events	Pollen	1.117	0.529	4.46	0.035
	Sugar	0.493	0.383	1.66	0.198
	Soybean	-0.780	0.854	0.83	0.361
Individual-based events	Pollen	1.723	0.912	3.57	0.059
	Sugar	-0.288	0.763	0.14	0.706
	Soybean	-24.078	195372	0.00	0.999
2: Color (with constant ingredients)					
All events	Color	N/A	N/A	23.00	<0.001
Individual-based events	Color	N/A	N/A	8.00	0.005
3: Ingredient (with constant color black)					
All events	Pollen	0.865	0.422	4.21	0.040
Individual-based events	Pollen	0.693	0.612	1.28	0.258
Relative brightness					
All events	Darker dough	-0.053	0.188	0.08	0.778
Individual-based events	Darker dough	-0.095	0.309	0.10	0.758
1a & 3: Olfaction and vision interaction					
All events	Yellow	3.258	1.019	10.22	0.001
	Black	-2.393	1.103	4.71	0.030
Individual-based events	Yellow	3.258	1.019	10.22	0.001
	Black	-2.565	1.189	4.65	0.031
Effect of color on visitation rate					
All visits	Proportion of yellow dough	2.402	0.367	42.89	<0.001

In experiment 2, we used a standard Chi-square test (see Methods). All other Chi-square values are Wald Chi-square values as calculated by SAS 9.4. Each Chi-square test has one degree of freedom.

### Experiment 2: varying color with constant ingredients

In all visits, this experiment showed an exclusive selection of yellow doughs (23 visits) over black (present during 18 visits) or green doughs (present during the remaining 5 visits) (Fig 3). Consequently, the Chi-square tests showed a significant deviation from the null hypothesis (Table 2).

### Experiment 3: varying pollen with constant color black

When all foraging events were considered, this experiment again showed a strong preference for pollen-containing doughs (19 events) over pollen-lacking doughs (5 events), with only three visits during which both types of doughs were chosen (Fig 4), which resulted in a statistically significant preference for pollen-containing doughs (Table 2). When only the individual-based foraging events were considered, the same preference was maintained (Fig 4) but the smaller sample size resulted in a non-significant statistical result (Table 2). Individuals thus exclusively chose pollen-containing doughs in 70.4% of visits (19 out of 27, Fig 4) when all events were considered and 66.7% of visits (8 out of 12, Fig 4) when only the individual-based events were considered.

### Effect of relative brightness on food choice

**Fig 5:**
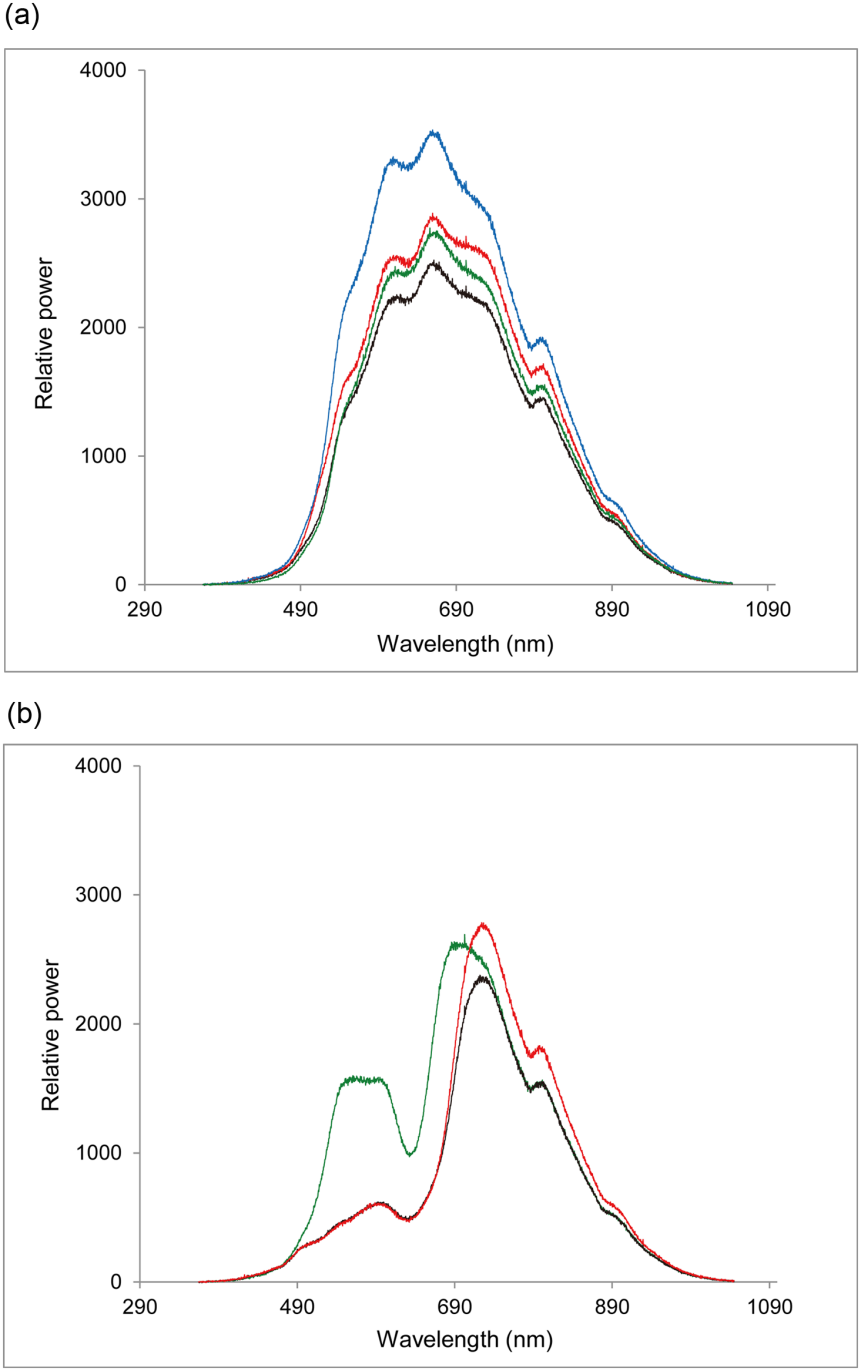
Spectra of reflected light by doughs in two-choice experiments. (a) Yellow dough with all three ingredients (black line); yellow dough without pollen (red line); yellow dough without soybean (blue line); yellow dough without sugar (green line). (b) Black doughs with all three ingredients (black line); black doughs without pollen (red line); green doughs with all three ingredients (green line).

The doughs’ reflected spectra clearly demonstrated that the three different colors resulted in three different spectra (Fig 5), but that yellow doughs with different ingredients had very similar spectra except for differences in their relative brightnesses. The black dough containing all three ingredients had the lowest brightness (area under the curve in Fig 5b) of all types of doughs. Setting its brightness as 1, the brightness of black dough lacking pollen was 1.13, green dough with all three ingredients was 1.38 (Fig 5b), original yellow pollen dough was 1.45, yellow dough lacking sugar was 1.55, yellow dough lacking pollen was 1.68, and yellow dough lacking soybean was 2.03 (Fig 5a). Therefore, the darker dough in each experiment was the original yellow pollen dough in experiment 1, the black or green dough in experiment 2 and the black dough with pollen in experiment 3. In experiment 1, the foraging events on the brighter and darker doughs were 34 and 62, respectively, when all foraging events were considered, and 8 and 21, respectively, when only the individual-based events were considered (Fig 2). In experiment 3, the foraging events on the brighter and darker doughs were 8 and 22, respectively, when all foraging events were considered, and 4 and 10, respectively, when only the individual-based events were considered (Fig 4). Therefore, we found no significant effect of relative brightness on the first chosen dough, regardless of whether we included all or only individual-based events (Table 2).

### Interaction between olfactory and visual cues

Combining the results from experiments 1a and 3, we found that the color yellow had a positive and significant effect on the probability of choosing a dough while the color black had a negative and significant effect, whereby the effect was stronger for yellow than black (Table 2). Finally, the proportion of yellow doughs had a positive and significant effect on the buzzard’s visitation rate (Table 2).

### Olfactory receptor gene analysis

**Fig 6:**
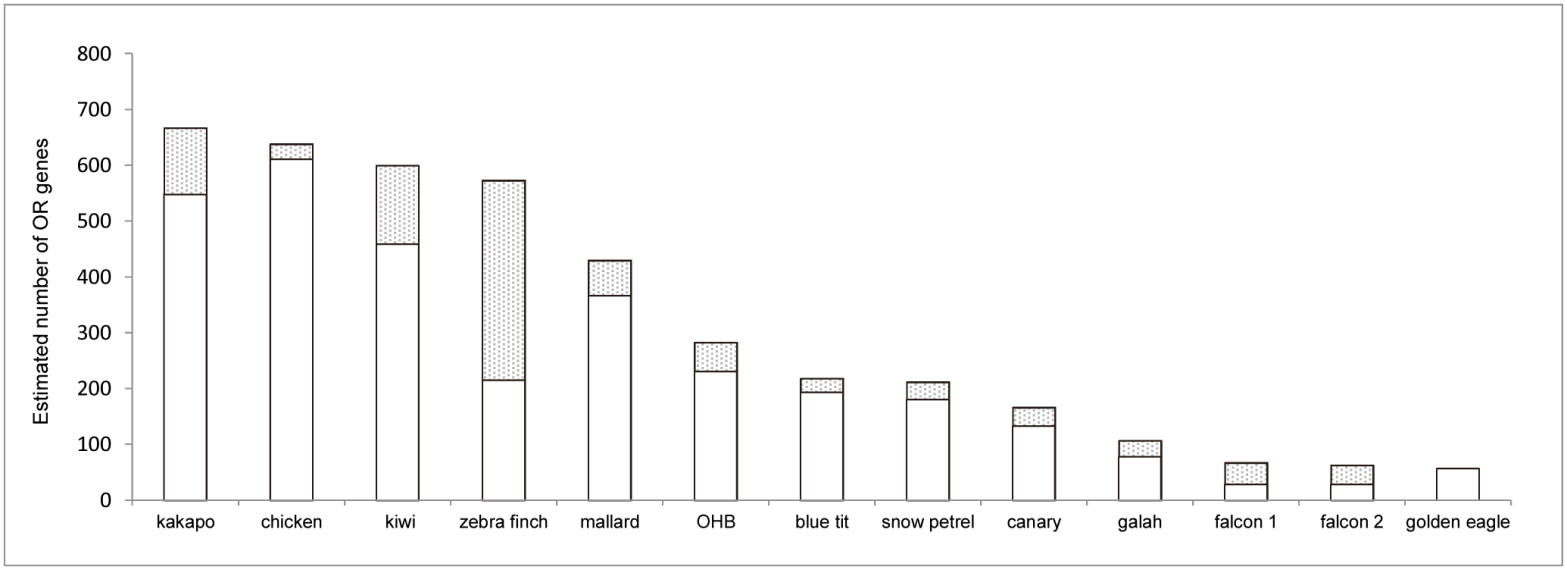
The estimated OR gene repertoire sizes of 13 species of birds representing eight orders. Open and grey bars represent the estimated total number of functional and non-functional OR genes, respectively. Data are from this study (OHB = Oriental honey buzzard), Warren et al. [41] (zebra finch), Zhan et al. [42] (falcon 1 = peregrine falcon, falcon 2 = saker falcon), Doyle et al. [43] (golden eagle) and Steiger et al. [38] (all other species). Note that Doyle et al. [43] did not distinguish between the number of functional and non-functional OR gene.

We obtained 39 and 26 distinct coding sequences from the partial γ-c and non-γ-c OR genes, respectively. Of these, 31 (79.5%) and 22 (84.6%) were functional, respectively. Thus, a total of 53 out of 65 (81.5%) of sequences were functional. The estimated total number of OR genes, including both functional and non-functional sequences, was 283. The OR gene repertoire sizes of the Oriental honey buzzard and other bird species are shown in Fig 6.

## Discussion

The first result of our experiments was that Oriental honey buzzards clearly selected pollen-containing doughs over pollen-lacking doughs. This choice was likely taken on the basis of different smells because we tested for the possible effects of color and relative brightness and concluded that the pollen-containing and the pollen-lacking doughs were almost visually identical in shape, color and brightness. Furthermore, it was clearly the pollen the buzzards were interested in, as no such preferential selection was observed for equal-looking doughs differing only in sugar or soybean content.

We further showed that the Oriental honey buzzard has a total number of OR genes smaller but relatively close to the average of 12 other species. However, among the diurnal raptors, the Oriental honey buzzard has by far the largest OR gene repertoire which is almost five times as large as the average for golden eagle (57), saker falcon (63) and peregrine falcon (67). Although our field experiments cannot completely exclude the possibility of buzzards choosing pollen-containing doughs for other yet unknown reasons, the existence of a relatively strong physiological basis of olfaction in the Oriental honey buzzard provides further support to the notion that the most parsimonious explanation of how buzzards distinguished doughs was via different smells. We concede that to conclusively proof the use of olfaction, we would need to visually hide doughs so that vision does not interfere at all with the experimental conclusions. However, if we concealed the doughs, we may have failed to attract the buzzards if their olfaction ability was not as acute as that of New World vultures. Further, when buzzards did not visit an experiment, we were not able to know whether buzzards were simply not around or whether they could not smell the doughs from where they were. Taking all our results together, we argue that, at least at close distances, the Oriental honey buzzard are likely to use olfaction when making food choices of foods with distinctive smells at close distances. To our knowledge, this is the first demonstrated case for a diurnal raptor to use olfaction which is not a New World vulture.

Interestingly, these food choices based on olfaction seemed to be further influenced by color vision. Several raptor species are known to have good color vision [44]. The Oriental honey buzzards also demonstrated their use of color vision when identifying food because (1) buzzards *always* preferred yellow pollen-containing doughs over black or green pollen-containing doughs, (2) the proportion of yellow doughs offered during each type of experiment was positively correlated with the buzzards’ visitation rate, and (3) when presented with only black colored doughs containing either pollen or not, buzzards would still prefer the pollen-containing doughs, but at a significantly lower rate than when presented with yellow doughs. Meanwhile, the relative brightness of the doughs did not affect the buzzards’ choices, excluding this potentially confounding factor. Therefore, we conclude that the buzzards’ color vision also influenced their foraging decisions whereby yellow was preferred over black and green. There may be two explanations for this preference which are not mutually exclusive: (1) Typical food items such as honeycombs are yellow, so buzzards may have an innate preference for yellow, or they could have an innate aversion towards the colors black and green; (2) some buzzards may have already been accustomed to yellow doughs which beekeepers had provided for many years (see Introduction) and were thus more likely to identify them as likely food objects or experienced neophobia when they encountered ‘novel’ black and green doughs. Further experiments repeated with the same individuals could distinguish whether individuals eventually lose their fear of novel colors or not, although this would almost certainly require captive individuals, giving the transience of wild buzzards in Taiwan (see Methods).

Our results therefore suggest the combined use of olfaction and color vision in the Oriental honey buzzard when making foraging decisions which involve foods with a distinctive smell, such as pollen, at close distances. Our results furthermore suggest that olfaction generally dominates over vision when the two senses convey conflicting information. The preference for pollen may have evolved along with the buzzard’s specialization of certain food items. Bees and wasps (usually larvae) form the main part of the buzzard’s diet [45]. During the breeding season, food items delivered by the parents to their young are mainly complete wasp nests (> 70%) [29, 31, 46]. Buzzards also prey on the nests of larger-sized, aggressive Hymenoptera species, such as bees and hornets [31], but they ignore paper wasp nests with a total biomass < 9 g [46]. Pollen, which is the major source of protein, lipid and minerals for honey bee larvae [47, 48] and many wasp species [49, 50], has a protein content reaching a maximum of 73.0% with an average of 39.8% [48] and is stored separately from the nectar cells inside the hive [51]. When foraging on bee hives, buzzards will inevitably consume the concentrated stores of pollen and thereby encounter its yellowish appearance. Buzzards may thus have learned or even evolved to identify this nutritious food item by both its smell and color.

The fact that the Oriental honey buzzard has an OR gene repertoire which is almost five times as large as that of three other raptors indicates that olfaction is of far greater ecological importance to the Oriental honey buzzard than other raptor species, except for some vulture species. For comparative purposes, studies of the OR gene repertoire of *Cathartes* vultures are hence called for. Even among all species, the OR gene repertoire of the Oriental honey buzzard is in the intermediate range, thus strongly suggesting that olfaction is indeed important to the Oriental honey buzzard. While most raptors are likely to rely mostly or exclusively on the visual and acoustic senses for prey detection, this may not be true for a few specialized species, such as honey buzzards and vultures, which may not be revealed without behavioral experiments and genetic analyses.

Our experiments may therefore be the first demonstrated case where olfaction was incorporated in a ‘multi-modal foraging strategy’ among raptors which was previously suggested for procellariiform seabirds [21, 22]. In our case, olfaction seems to be dominant over vision because, even for black doughs, the buzzards more often chose the pollen-containing than the pollen-lacking dough (experiment 3). Given that Lind et al. [44] stated that “the role of color vision in raptor foraging remains unclear,” our experiments may also be among the first ones to demonstrate an effect of color vision on raptor foraging decisions.

Although our experiments only demonstrate the interactive use of olfaction and vision at very short distances, it is possible that the Oriental honey buzzard locates food sources such as apiaries or natural bee hives over larger distances using either or both senses, e.g., smelling pollen at distances of a few hundred meters or even a few kilometers, or seeing bees fly in concentrated numbers above the canopy from further away. Vultures certainly use olfaction to detect food which is hidden from sight at large distances (e.g., [5]). Since our food was not hidden from sight, we have not tested yet whether Oriental honey buzzards also use olfaction for food detection over larger distances or when food is hidden. However, given that many natural food sources of honey buzzards tend to be well hidden from sight (e.g., bee hives hidden inside tree trunks or underground), this possibility should certainly be tested by visually hiding pollen-containing food items. Future studies could also use telemetry to investigate whether olfactory or visual detection works at larger distances.

In conclusion, our field experiments combined with our genetic analyses support the notion that Oriental honey buzzards use a multi-modal foraging strategy which probably involves both olfaction and vision when choosing food items containing pollen or not which are directly in front of them. Because of their specialization on visually hidden foods provided by Hymenoptera, olfaction should have evolved to become more important to honey buzzards. To more conclusively demonstrate the use of olfaction, future studies should provide only the smell of pollen or other food items without providing any visual cues. We know olfaction is also important to vultures (see Introduction), but we do not know whether other diurnal raptors use olfaction, and if, to what degree. Since olfaction may play some role in the behaviors of many or even all bird species, we suggest that olfaction should be considered in the design of behavioral and genetic studies to better understand the use of multiple senses in the behavioral processes of birds.

## Supporting Information

S1 File Coordinates of eight apiaries for field experiments. (DOCX)
