# Supplementary material for: Stop and Smell the Pollen: The Role of Olfaction and Vision of the Oriental Honey Buzzard in Identifying Food
Source: PLoS One. 2015 Jul 15;10(7):e0130191. doi: 10.1371/journal.pone.0130191 (PMC4503435; doi:10.1371/journal.pone.0130191)
Supplement: S1 File — (DOCX) [file pone.0130191.s001.docx]

S1. Names and coordinates of the eight apiaries where choice experiments were conducted in central and southern Taiwan.

|  | Name | Coordinates |
| --- | --- | --- |
| Apiary 1 | Jiufengshan | 23.969°N, 120.787°E |
| Apiary 2 | Jiulongshan | 22.042°N, 120.825°E |
| Apiary 3 | Niujiaowan | 22.642°N, 120.621°E |
| Apiary 4 | Hejie | 21.956°N, 120.714°E |
| Apiary 5 | Zhonglin | 22.650°N, 120.604°E |
| Apiary 6 | Manzhou | 22.016°N, 120.836°E |
| Apiary 7 | Longquan | 21.985°N, 120.727°E |
| Apiary 8 | Guanshan | 21.974°N, 120.725°E |
